# Comparing Video Analysis to Computerized Detection of Limb Position for the Diagnosis of Movement Control during Back Squat Exercise with Overload

**DOI:** 10.3390/s24061910

**Published:** 2024-03-16

**Authors:** André B. Peres, Andrei Sancassani, Eliane A. Castro, Tiago A. F. Almeida, Danilo A. Massini, Anderson G. Macedo, Mário C. Espada, Víctor Hernández-Beltrán, José M. Gamonales, Dalton M. Pessôa Filho

**Affiliations:** 1 Instituto Federal de Educação, Ciência e Tecnologia de São Paulo (IFSP), Piracicaba 13414-155, SP, Brazil; andreperes@ifsp.edu.br; 2 Graduate Programme in Human Development and Technologies, São Paulo State University (UNESP), Rio Claro 13506-900, SP, Brazil; andreisanca@hotmail.com (A.S.); elianeaparecidacastro@gmail.com (E.A.C.); tiagofalmeida.w@gmail.com (T.A.F.A.); dmassini@hotmail.com (D.A.M.); andersongmacedo@yahoo.com.br (A.G.M.); 3 Department of Physical Education, School of Sciences (FC), São Paulo State University (UNESP), Bauru 17033-360, SP, Brazil; 4 Pos-Graduation Program in Rehabilitation Sciences, Institute of Motricity Sciences, Federal University of Alfenas (UNIFAL), Alfenas 37133-840, MG, Brazil; 5 Instituto Politécnico de Setúbal, Escola Superior de Educação, 2914-504 Setúbal, Portugal; mario.espada@ese.ips.pt; 6 Sport Physical Activity and Health Research & INnovation CenTer (SPRINT), 2040-413 Rio Maior, Portugal; 7 Centre for the Study of Human Performance (CIPER), Faculdade de Motricidade Humana, Universidade de Lisboa, 1499-002 Cruz Quebrada, Portugal; 8 Comprehensive Health Research Centre (CHRC), Universidade de Évora, 7004-516 Évora, Portugal; 9 Life Quality Research Centre (CIEQV-Leiria), 2040-413 Rio Maior, Portugal; 10 Training Optimization and Sports Performance Research Group (GOERD), Faculty of Sport Science, University of Extremadura, 10005 Cáceres, Spain; vhernandpw@alumnos.unex.es (V.H.-B.); martingamonales@unex.es (J.M.G.); 11 Facultad Ciencias de la Salud, Universidad Francisco de Vitoria, 28223 Madrid, Spain; 12 Programa de Doctorado en Educación y Tecnología, Universidad a Distancia de Madrid, 28400 Madrid, Spain

**Keywords:** pattern recognition, motor activity, computer modelling, strength training

## Abstract

Incorrect limb position while lifting heavy weights might compromise athlete success during weightlifting performance, similar to the way that it increases the risk of muscle injuries during resistance exercises, regardless of the individual’s level of experience. However, practitioners might not have the necessary background knowledge for self-supervision of limb position and adjustment of the lifting position when improper movement occurs. Therefore, the computerized analysis of movement patterns might assist people in detecting changes in limb position during exercises with different loads or enhance the analysis of an observer with expertise in weightlifting exercises. In this study, hidden Markov models (HMMs) were employed to automate the detection of joint position and barbell trajectory during back squat exercises. Ten volunteers performed three lift movements each with a 0, 50, and 75% load based on body weight. A smartphone was used to record the movements in the sagittal plane, providing information for the analysis of variance and identifying significant position changes by video analysis (*p* < 0.05). Data from individuals performing the same movements with no added weight load were used to train the HMMs to identify changes in the pattern. A comparison of HMMs and human experts revealed between 40% and 90% agreement, indicating the reliability of HMMs for identifying changes in the control of movements with added weight load. In addition, the results highlighted that HMMs can detect changes imperceptible to the human visual analysis.

## 1. Introduction

Resistance training (RT) is an exercise that has grown in popularity in recent decades and has been shown to be effective in improving athletic performance, preventing injuries, and playing a major role in muscle conditioning and body weight programs [1,2,3]. Although the sport- and health-related effects of RT are well-documented [4,5], 25–30% of individuals who practice RT are at risk of injury [6,7]; the factors that increase injury prevalence include unsupervised training, uncontrolled management of load intensity, and inappropriate techniques [8].

Most assessments conducted by coaches are based on the observation of movements performed during physical activities [9]. It is important for RT practitioners to monitor their movements during the exercises to optimize benefits and reduce the risk of injury. A precise method for detecting changes in motor activity patterns can play an important role in this process, and automating the method could provide several benefits [10]. For an adequate intervention, an effective assessment that is capable of identifying the deficits in the practitioner’s activity is necessary. The mathematical modelling of movements could contribute significantly to the recognition of postural changes during exercise.

Hidden Markov models (HMMs) are statistical models with great applicability and efficiency in pattern recognition because they adapt well to the analysis of sequential data; for this reason, HMMs are considered the state of the art in modelling this type of data [11]. HMMs are used to recognize movements made by objects or humans in many studies, some of which utilize the segmentation of sequential motion images [12,13,14].

Data captured by accelerometers and gyroscopes are also used to model movement with HMMs [15]. Depth sensors that use pixels from different layers to generate and recognize images are currently used for modelling coordinates. Microsoft^®^ Kinect sensors, for example, are widely used to capture motion coordinates in research [16,17,18,19].

Previous studies provide relevant research on the use of Cartesian coordinates for modelling human movements with HMMs. Some studies have used the full-body motion of humans to analyse their trajectory within a limited space [20,21,22], while others have used movements performed only by body segments [23,24,25]. Research in technology-related areas [23,26,27,28] and health care areas [25] recommend using HMMs powered by two-dimensional coordinates in situations where the ability to model movement and its accuracy are essential.

With the popularization of technology, video capture equipment has become increasingly affordable and technologically robust. Cameras have been used for video analysis [21,29] and are noninvasive, do not interfere with the execution of the exercise, and enable the collection of information for the analysis of physical activity or movement, addressing the limitations of qualitative analysis by direct human observation [30]. Current smartphones have cameras with an image resolution that used to be found only in professional equipment. Processing movement videos using automatic tracking software provides joint positional data during the activity, and these positional data can be mathematically modelled to provide motion characteristics.

In the current study, cartesian coordinates of joint trajectories during the performance of back squat exercises were used to identify changes in the movement pattern caused by an increased load. The back squat exercise was chosen because it is commonly practiced to enhance the strength of lower limb muscles (i.e., quadriceps, hamstring, gastrocnemius, and gluteus maximus) in both conditioning and rehabilitation programs [31,32]. Previous studies, reported that knee joint horizontal displacement is noticeable when squatting with a load at 50% of an individual’s body weight, and that the coordination pattern of knee position differed according to sex and performer experience [31]. Pattern disarrangement during the descent phase of the squat exercise with weight loads is well-reported, suggesting that the vertical displacement (i.e., the ability to lift the load during the ascent phase), another important component of the squatting pattern, might also be impaired, mainly when performing the exercise with heavier loads. This theory has not been addressed, so the current study aimed to verify if back squat vertical movement is altered when heavier weight is added and if current computerized technology is suitable for this analysis. Therefore, the potential of HMMs to identify changes in movement control when performing back squats with progressively increasing loads (addition of 50 and 75% of body weight) was investigated. Human experts also visually analysed the execution of back squat exercises with and without loads, and the results of HMM tracking recognition and human observation were compared in a qualitative analysis. The hypothesis is that computerized HMM analysis can identify pattern alterations captured with 2D images that are not perceived by visual analysis.

## 2. Materials and Methods

### 2.1. Participants

The sample consisted of ten male volunteers, aged 26.3 ± 4.9 (mean ± SD) years, with a height of 177.6 ± 8.0 cm and a body weight of 86.2 ± 16.7 kg. All participants had a minimum of six months of experience performing weightlifting exercises. The Human Research Ethics Committee of the local university approved the research (CAEE: 17486119.0.0000.5398).

### 2.2. Data Collection

Data collection was carried out at the Human Sports Performance Optimization Laboratory (LABOREH) at UNESP, Bauru Campus. Green semispherical markers were affixed to the ankle, knee, and hip of the volunteers, and to the barbell for execution of the back squat exercise.

The volunteers performed three complete repetitions of the back squat exercise with no load added to the barbell (i.e., performing the descent and ascent carrying only the barbell weight [~11.3 kg] and the whole-body weight), which was considered the reference for the back squat movement since no movement constraints are expected for experienced and uninjured persons under these conditions [31]. Immediately after a 10 min break, they performed three more repetitions of the loaded exercise (using the barbell) with 50% of their body weight, and after another break, they performed three more repetitions with a load of 75% of their body weight. The increased load was used to cause movement disarrangement.

**Figure 1:**
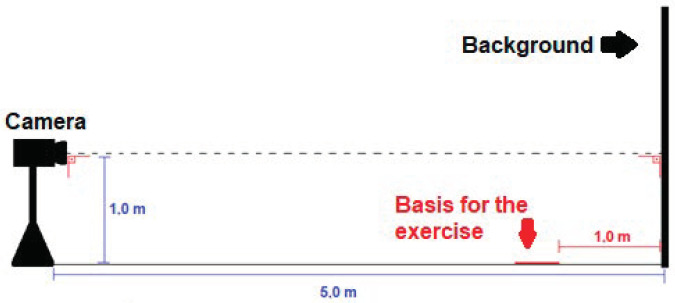
Scheme for capturing videos.

To collect the positional data for the back squat exercises, a digital video camera coupled to a Samsung^®^ smartphone model Galaxy S9 (12 megapixels with 4K UHD resolution 3840 × 2160, Seoul, Republic of Korea) was used. Data were collected with the camera in a stationary position, with the optical axis perpendicular to the participant’s sagittal plane, 1 m from the ground, and 5 m from the background (Figure 1) [33].

**Figure 2:**
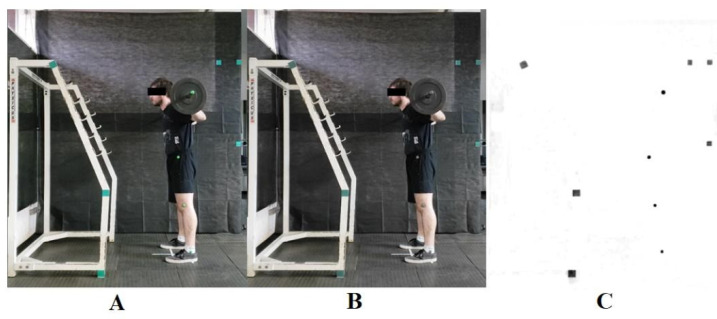
Example of filters applied to videos of back squat exercise. (**A**) Original video image. (**B**) Application of the Chroma Key effect, which locates and changes the colour of the markers. (**C**) Application of the Alpha channel, which subtracts everything that was not selected by the Chroma Key.

Calibration to the environment in which the back squat exercises were performed was determined from the distances between the markers on the background and the plane that divides the participant’s body symmetrically into two sides: right and left (sagittal plane) (Figure 2). Using this procedure, it was possible to measure the displacement of the two-dimensional (2D) coordinates of the hip, knee and ankle joints during the back squat in the appropriate plane. Videos of three complete executions of the proposed exercises for each of the three load variations were captured in MPEG-4 format at a frequency of 30 frames per second.

**Figure 3:**
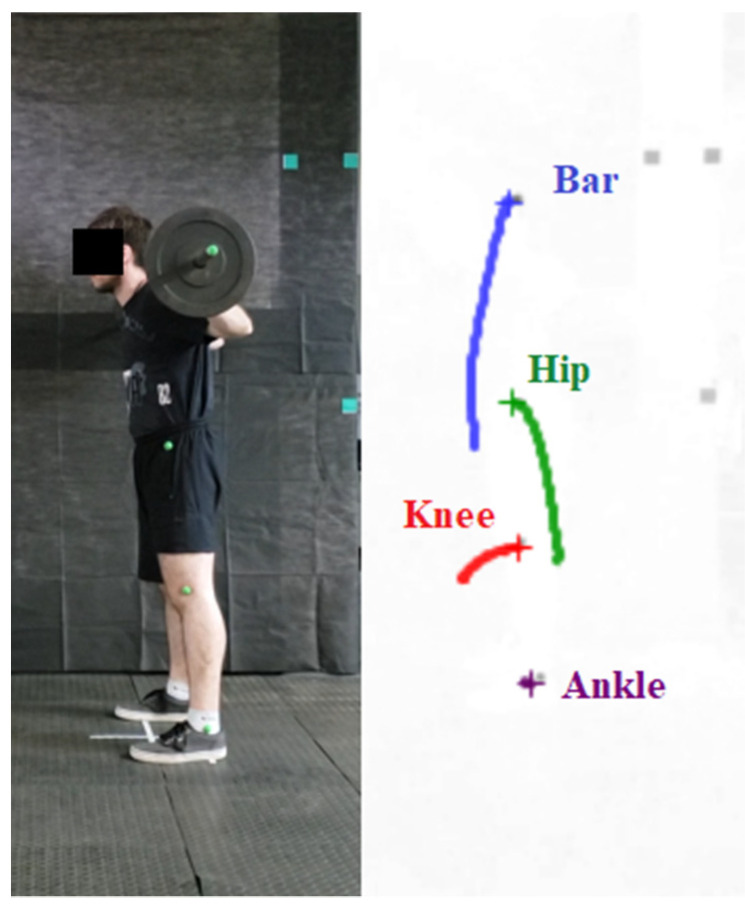
Trajectory of each joint during a back squat exercise. Tracking was performed with Kinovea software (version 0.8.27, Bordeaux, France).

Digital processing of the videos was carried out with the video editor Wondershare Filmora (version 9, Hong Kong, China); the Chroma Key effect was applied to the colour of the markers, and the Alpha channel was used to better contrast the markers with the surroundings (Figure 2). Kinovea software (version 0.8.27, Bordeaux, France) was used for semiautomatic tracing of the markers. The origin of the coordinates in the Cartesian plane (i.e., an ordered pair (x, y) representing the position on the horizontal x-axis from left to right and on the vertical y-axis from bottom to top) was assigned to the markers placed on the hip, knee, ankle, and barbell, which were recalibrated for each of the repetitions (Figure 3).

Studies have confirmed the validity and accuracy of using Kinovea software (version 0.8.27, Bordeaux, France) in video a nalysis to measure the kinematic variables of the limbs in the frontal and sagittal planes in different sports, including weightlifting [34,35,36,37]. In addition, video analysis using Kinovea software (version 0.8.27, Bordeaux, France) is highly accurate and reliable when compared to other procedures for measuring athletes’ jumps (~3.1 mm of error) [36].

### 2.3. Displacement and Vertical Distance Measurements

Joint displacement measurements were obtained from the markers placed at the iliac crest (hip), femoral lateral epicondyle (knee), lateral malleolus (ankle), and the barbell (see Figure 1), according to the following formula for Euclidean distance calculation:

(1) $$d=\sqrt{{\left(x_{f}-x_{i}\right)}^{2}+{{(y}_{f}-y_{i})}^{2}}$$

where d is the displacement value, (*x_f_*, *y_f_*) are the coordinates of the markers at the final position, and (*x_i_*, *y_i_*) are the coordinates of the markers at the initial position.

The variation in the vertical distance of each marker in relation to the y-axis, considering x values equal to zero, was calculated with the following formula:

(2) $$\Delta y=y_{f}-y_{i}$$

where Δ*y* is the vertical distance, $y_{f}$ is the y-value of the coordinate at the end point, and $y_{i}$ is the y-value of the coordinate at the starting point. Only the ascent phase of the back squat was analysed since the aim of the mathematical modelling was to be an analytic tool to evaluate the ability of an individual to lift a given load, which utilizes the contractile properties of muscle to overcome resistance and control sources of joint instability, therefore ensuring safety and efficacy in training and rehabilitation [31,32].

### 2.4. Modelling with HMMs

Based on Markov chains, HMMs have a finite number of states and utilize a double stochastic process: a transition between states and an output symbol generated for each state, both of which are linked to a probability of occurrence. In HMMs, the hidden sequence of states can be estimated by the visible observation sequence [38], which in our case is the spatial position at every moment of a movement. Symbolically, the models can be characterized by the following elements [39]:

- *N* represents the number of states in the model;
- *S* = {*S*_1_, *S*_2_, …, *S_N_*} corresponds to the set of individual states in the model;
- *M* represents the number of distinct observations by state;
- *O* = {*o_k_*}, *k* = 1, …, *M* corresponds to the set of individual observations;
- *A* = {*a_ij_*} corresponds to the distribution of transition probabilities of states and is calculated as follows:
  (3) $$A=\left\{a_{ij}=P\left({\;S}_{i\;}a\;t\right)\right\},\;t=1,\ldots ,T.$$
  Thus, the probability that the model moves to state *Sj* at time *t* + 1 depends only on the state *S_i_* at time *t*, which is characteristic of a Markovian model.
- $B=\left\{b_{ik}\right\}$ corresponds to the probability distribution of the observation in each state and is calculated as follows:
  (4) $$B=\{b_{ik}={b_{i}(O}_{k})=P\left({\;S}_{i}\right)\}$$
- $\pi =\left\{p_{i}=P(S_{i}\;a\;t=1)\right\}$ corresponds to the initial distribution of states;
- *λ* represents the model given by *λ = {A, B, π}*.

To apply HMMs, three basic problems must be solved: assessing the probability of the unknown sequence, estimating the best sequence of hidden states, and training the model parameters. In the model learning phase, the training data are divided into groups (clustering). Training is performed based on maximum likelihood using the Baum–Welch algorithm [40], which provides the most likely transition probabilities and the most likely set of emission probabilities, considering only the observed states of the model. In the Baum–Welch algorithm, from an initial estimate λ of the model and O of the observation sequence, the best estimates of the parameters of a new model *λ^NM^* are obtained as follows [41]:

(5) $$a_{ij}^{NM}=\frac{P(transition\;from\;q_{i}\;state\;to\;state\;q_{j}/O,\lambda )}{P(transition\;from\;q_{i}\;state\;to\;any\;state/O,\lambda )}$$

(6) $$b_{jk}^{NM}=\frac{P(emission\;of\;the\;v_{k}symbol\;to\;the\;state\;q_{j}/O,\lambda )}{P(emission\;of\;any\;symbol\;from\;the\;state\;q_{j}/O,\lambda )}$$

(7) $$\pi_{i}^{NM}=P(observation\;sequence\;starting\;at\;the\;state\;q_{i}/O,\lambda )$$

After model formation, the recognition process is performed to choose the model that provides the maximum probability of the analysed observation sequences P(O|λ) [42]. Formulas (5)–(7) guarantee the addition of P(O|λ) until a convergence point is reached, at which point there is no additional variation in the parameters.

The general topology used for modelling by HMMs is linear [43], where each state connects to itself and to the next state and there is no transition from the last state to the first state [44]. Only the ascendent movement of the exercise was analysed. The Markovian model algorithms used for this experiment [45,46,47,48] were implemented in GNU Octave 4.2.1. For analysis, two-dimensional data were provided to the HMMs regarding the trajectories of each joint during the ascending phase of the exercise.

**Figure 4:**
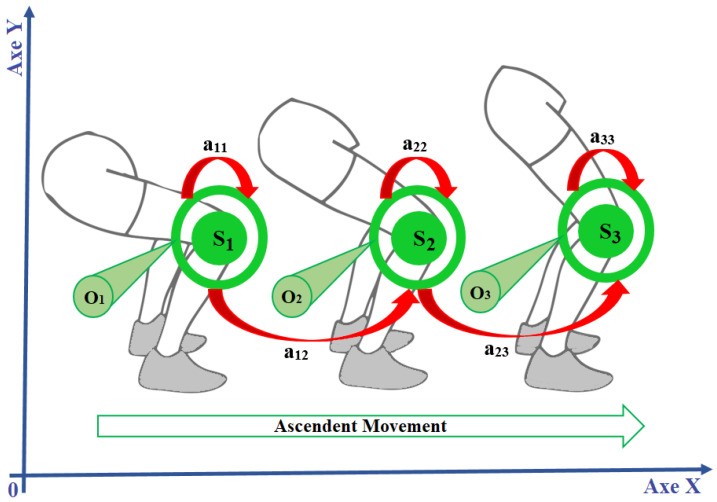
Relationship between bidimensional positions of the knee during movement with Markovian modelling in the upwards phase of the back squat exercise.

For learning, the data were clustered into groups [29] using the K-means algorithm [49], which generates the states of the HMMs. The k-means cluster formation process is performed using an unsupervised procedure that seeks to minimize distance measures [50]. Figure 4 shows an example of a sequence of three scenes from the relationship between knee positioning during the ascendant movement of the exercise and the form of Markovian modelling that was performed. Each of the three knee positions, defined as states S_1_, S_2_, and S_3_ (trajectory clusters), shows the possibility of stopping the movement or its sequence according to the transition probabilities *a_ij_*. The Figure 4 also shows the probability of observation for each state *O_k_*.

For intrasubject modelling by HMMs, the first three executions without load (barbell only) were used as the training baseline for each participant. A movement carried out without a load is believed to have fewer errors than a movement modified by a load. The movement performed with an average load was also modelled to compare the movement changes with the maximum load. In this modelling step, the model parameters λ = (A, B, π) were calculated using the observed symbols O (Cartesian coordinates), so that P(O|λ) was maximized. The Baum–Welch algorithm was used, which maximizes the probability of the observation sequences [29,51].

The modelling of the exercise executions described by the marker trajectories was based on the criterion of highest similarity. To evaluate the similarity between a trained model and a new observation sequence (trajectory from another execution), the forwards algorithm was used [40], which uses the probability of being in each of the states and calculates the probability of the next time step immediately after. Afterwards, with the HMM, the minimum number of states needed to recognize the differences (displacements) was obtained. These differences were identified by variance analysis in the execution of the exercises due to the variation in the load.

### 2.5. Human Evaluation

Two evaluators, with over 30 years of experience, observed the performance of the exercise performed by the volunteers through videos as the observational reference in the data provided for the statistical/mathematical models. The evaluators visually analysed the differences in joint displacements by the trajectory of the markers with increasing loads. They used the exercise performed without the addition of a load as a reference. In comparisons of the exercises containing two different loads, they tried to identify any alteration in the pattern that could compromise the performance of the exercise. The evaluators compared the following points during the exercises: knee and hip at 0–50% and 0–75% load. The results of their evaluations were compared with those derived by ANOVA from the mathematical models using percentages of answers in agreement and interrater reliability statistics utilizing Cohen’s kappa coefficient.

### 2.6. Statistical Analysis

Statistical analysis was performed using the IBM SPSS Statistics 26.0 (SPSS, Chicago, IL, USA) program version 18.0.0. One-way analysis of variance (ANOVA) was performed to determine significant differences in the maximum displacement for each joint according to the different loads used during the ascending phase of the exercises. Data normality was confirmed with the Kolmogorov–Smirnov test before ANOVA testing. The results ensured the normality for hip, knee, and barbell distance data. Tukey’s post hoc test with a significance level of *p* < 0.05 was used. For the results from the human evaluators, the percentages of agreement between the statistical model and each evaluator and between evaluators were analysed. A statistical analysis of interrater reliability was also performed using Cohen’s kappa coefficient [52].

## 3. Results

The displacement values of each joint in each of the three executions during the ascending phase of the back squat exercise are shown in Table 1.

With the maximum displacement values for each marker, ANOVA was performed to determine significant displacement differences with the three different loads for the groups in the back squat exercises. However, no significant differences (ANOVA) were found in the displacement for any of the joints when analysed across groups. A new ANOVA was performed for displacement differences in each participant. The markers that showed significant differences in displacement for most participants (50% or more) were analysed by HMMs. Only markers at the knee and hip fit this criterion.

ANOVA identified joint position disturbances when comparing the 0 vs. 50% (five volunteers) and 0 vs. 75% (eight volunteers) conditions for the knee (*p* = 0.03) and the 0 vs. 50% (six volunteers) and 0 vs. 75% (six volunteers) conditions for the hip (*p* = 0.02). For comparisons that showed significant differences, models with a minimum number of states were created (Table 2). For the 0 vs. 50% comparison, n > 40 (50% IPC) for the knee and n > 45 (60% IPC) for the hip were identified. For the 0 vs. 75% comparison, n > 30 (80% IPC) for the knee and n > 40 (60% IPC) for the hip were identified.

To determine whether the generated models could identify movement differences in the execution of the exercises according to the change in load, only the executions of participants who showed significant differences in movement according to the analysis of variance were modelled.

By testing all the models, the largest number of states per load was found for each joint, and 100% identification of the differences in displacements between all participants that presented significant differences was achieved.

The values obtained for the displacements of each joint for each of the three executions during the ascending phase of the back squat exercise with a barbell are shown in Table 2.

**Figure 5:**
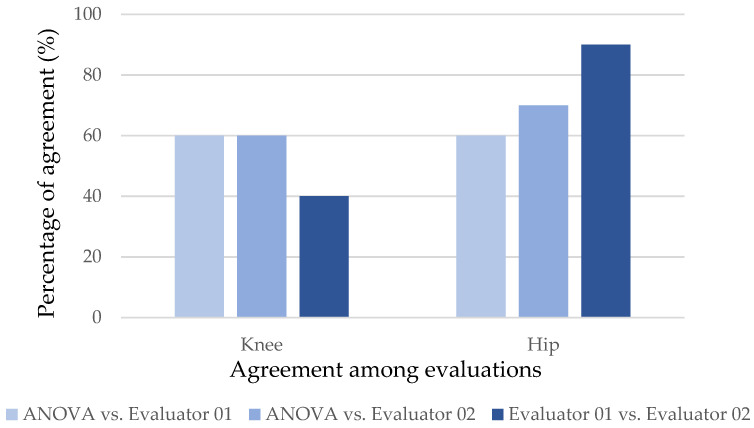
Graph of the percentages of agreement between responses identifying the displacement significance for the back squat exercise for the comparison of the 0–50% loads.

**Figure 6:**
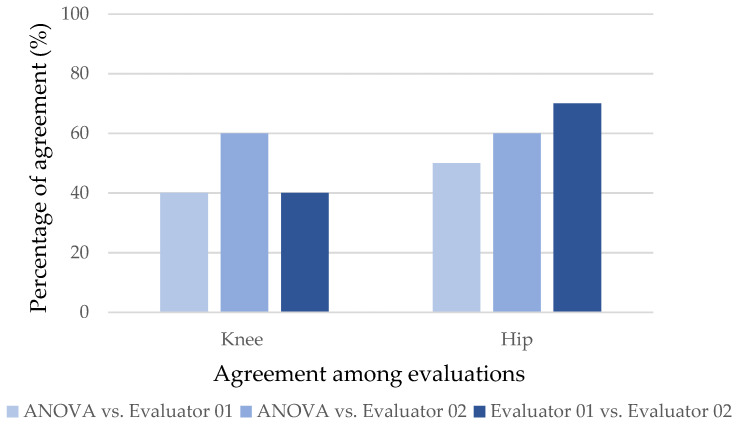
Graph of the percentages of agreement between responses identifying the displacement for the back squat exercise for the comparison of the 0–75% loads.

The human evaluators determined significant differences in joint displacement for the exercise (reference condition with the barbell only). The percentages of agreement between the statistical model and evaluators and between both evaluators are shown in Figure 5 and Figure 6.

The graphs show that the evaluators could identify the significance of the displacements indicated by ANOVA more frequently at a load of 50% (intermediate). More concordant results between both the ANOVA and the two evaluators and between the evaluators were found for the hip.

Kappa values were calculated for the back squat exercise at the 0–50% and 0–75% load ratios. Only the agreement between evaluators for the hip at the 0–50% ratio was significant (k = 0.783 and *p* = 0.011). For the 0–75% ratio, no statistically significant agreement was found.

## 4. Discussion

The objective of this study was to assess the value of HMMs in the identification of alterations in patterns during the back squat exercise due to an increase in load. A video camera was used for noninvasive data capture without the need for wearable equipment. As shown in the study by VanWye and Hoover [30], the use of video analysis has advantages compared to face-to-face qualitative analysis of movement because disturbances may not be perceptible to the human evaluator due to the speed of execution of the movement. Mckean et al. [31] reported changes in hip and knee movement patterns (angles) during squats, which was consistent with the current observations of knee and hip displacements during back squat exercises with loads at 50 and 75% of body weight compared with a motion reference. In addition, a previous study aiming to automate the movement pattern analysis of bicep curls using HMM algorithms reported that HMMs could detect joint positional adjustments during lift attempts with heavy loads, which showed no correspondence to the standard bicep curl [48]. Thus, the novelty of the current study was the evidence that an HMM is also a suitable computerized method to analyse more complex movement (i.e., multi-joint or multi-segmental motions) than a single-joint action, as analysed previously by Peres et al. [48]. Moreover, the current analysis demonstrated that HMMs can provide more accurate information on motion patterns than human visual observation, which was only speculated from the results of Peres et al. [53].

Moreover, Pueo et al. [54] used videos captured by smartphones to analyse the execution of squat exercises and reported that the distortions of smartphone cameras are negligible. The authors aimed to measure the position of the barbell during the exercise to derive the range of motion and speed of execution. As in our methodology, they used tracking software to automatically provide marker position data.

Regarding the Markov modelling applied in this work, the use of linear topology in HMMs [21,29,43] and the impediment of transition from the last state to the first state, as in the work of Nguyen et al. [44], was sufficient to understand the movements performed and the pattern changes in the execution of the exercises. Relating the Cartesian coordinates to the observable HMMs, these coordinates describing spatiotemporal ordering, with no possibility of jumping between states or indentations, strengthened the choice of topology employed. Using a two-dimensional model with data captured by a smartphone camera provided necessary and sufficient data for modelling by the HMMs, which identified the movements that showed significant differences according to the ANOVA results. Finding the minimum value of the number of states (N) for the recognition of the change indicates probabilistic convergence of the model for change detection. A second relevant aspect was creating a training base for the model. This was achieved by defining the correct movements, capturing data, and using HMMs as a comparison model.

No significant group differences were found. The participation of different muscles and joints [55] in movement execution, as well as the fact that the load is very close to the body, may have mitigated the need to maintain balance, making joint movements more restricted. Significant trajectory changes were found for two joints: the knee and hip. For these cases, HMMs were used for modelling, and the minimum number of states were identified to recognize the differences between the movement patterns.

Using the minimum number of states found, an intrasubject [56] model was developed that could recognize differences in executions, which may indicate that the maximum volume of individual load needed to change the execution pattern is appropriate. The relationship between the significant range of motion of the hip together with that of the knee confirms the change in the movement pattern due to the increased load. In all patients with a significant increase in the trajectory of the hip, there was also a significant increase in the range of motion of the knee (0% vs. 75%), and in 80% of patients with a significant increase in the trajectory of the hip, there was also a significant increase in the range of motion of the knee (0% vs. 50%). It is possible that there was a muscle readjustment to maintain balance, requiring the participation of more muscles [57,58,59], which led to altered positioning of the hip and knee, as identified in most cases.

For executions performed with a load of 50% of body weight, the evaluators achieved 60% to 70% agreement with the statistical model, but there was disagreement between the professionals regarding knee displacement (40%). For the hip, both evaluators had consistent agreement in their observations (90%). With the load change to 75% of body weight, there was less consistency of responses between the evaluators and the statistical model and between the evaluators. The concordant responses between the evaluators and the statistical model did not exceed 60%, and between the evaluators, there was a low agreement rate for the knee observations (40%).

In the comparison of the results obtained by the mathematical model and human evaluators, there was a good percentage of agreement both between the evaluators and the model and between the evaluators for some cases. Good agreement between evaluators indicates consistency in the perception of error in the human assessment [60]. Greater inconsistency between responses was observed for the joint with less instability, the knee. This result for the knee joint can be attributed to its lower range of joint oscillation during the movement trajectory when compared to the hip and, therefore, less noticeable unbalance to the human eye. These results are consistent with those of Porz et al. [60] regarding the need to use a computational tool to obtain a better diagnosis.

The identification of significant trajectory differences by ANOVA confirms the occurrence of movements with no correspondence to the established reference, which are not always identified by human visual observation. The work of Fang et al. [61] showed that not all attributes are perceptible to a human observer, so the computational model can surpass human ability in some cases. This statement is consistent with the assumption that the human visual observation of movement is based on low spatial accuracy and temporal resolution [62], therefore emphasizing the importance of the method developed in the current study, by which different joints can be tracked and analysed simultaneously. The HMMs managed to perfectly model the cases of motion disarrangement indicated by the statistical treatment, achieving a high recognition rate in participants with significant differences. HMMs can more accurately and quickly identify changes in movement patterns that are not perceptible to human visual observation [63], which may have occurred due to inattention or because they are not as relevant to the evaluator.

Similar to the work by Porz et al. [60], a comparison was made between the computational tool and human evaluators, and kappa interrater agreement analysis was also performed to verify the agreement between the model and human evaluators and between the human evaluators. The only statistically significant difference in the kappa coefficient was found between human evaluators for the hip in the 0–50% of body weight comparisons. Comparing the percentage of consonance between the model’s responses and the responses of the evaluators and between the evaluators, it was observed that the kappa statistic did not represent the agreement between them very well. This can be seen in comparisons that reached 60% agreement (knee at 50% of body weight) between the model and the human evaluators, but the kappa statistic did not reach significance (k = 0.200 and *p* = 0.292 for evaluator 01 and k = 0.200 and *p* = 0.527 for evaluator 02).

However, unlike the HMM, visual observation is an estimation process in which no body model is available as a reference, and a direct relation between image observation and the silhouette of motion reference must be established [62,63]. Indeed, if the reference strongly influences what features are observed, the level of correspondence tends to be associated with the accuracy of the cognitive representation of the movement pattern to be analysed [64]. Hence, considering that perception is an important aspect of movement analysis by visual observation, the results of kappa coefficients (above 0.4) suggest moderate to substantial levels of agreement between observers, which is qualitatively acceptable [65]. The method developed has a limitation in that it only analyses movement displacement in a two-dimensional (2D) manner. By expanding the analysis to three dimensions (3D), more detailed information on motion in other planes of movement can be obtained, providing insights into changes in joint position that may occur to maintain postural adequacy with increased load. The 3D sagittal model provides more accurate data and is not subject to the parallax and perspective errors that occur in 2D analyses. However, the back squat exercise movement is primarily in the sagittal plane rather than in the transverse and frontal planes [66]. Although the 3D approach has advantages over the 2D approach, the 2D analyses, such as the one described in the study, allow the analysis of motion using data collected in any type of video. However, the use of HMM analysis with 3D data could identify additional changes caused by an increase in load, providing more information about changes in movement trajectories through simultaneous modelling in different planes [67].

Moreover, while the selected sample of participants (ten males) supported the development of the HMM to analyse of back squat execution, additional limitations of the current model are its application to the analysis of sex-related particularities during squatting execution or its variants (e.g., trunk alignment, ankle-knee-hip kinematic chain, knee forward movement in sagittal plane, and squat depth) [33,68,69,70] and the application of the HMM to the analysis of other mechanical features of exercise execution, such as the ability to perform (e.g., novice vs. advanced) [71], and the level of motor disability during the movement (e.g., neural dysfunctions) [72], since all these conditions require a large sample of participants to achieve an ideal reference for each movement condition for HMM analysis. On the other hand, evaluators performing visual observation of the performance might perceive (and evaluate) this variation in back squat execution as incorrect due to the lack of an appropriate reference or even perceive small functional motion variability as disarrangements [62,63]. To avoid additional sources of misinterpretation in the analysis of the movement by evaluators, the current study recruited a single group of experienced male lifters, but even in this category of participants (i.e., advanced), there are qualitative motor differences during the execution [33,73] that might contribute to the disagreement of judgement between evaluators. Finally, future research should focus on analysing subjects’ movements in three dimensions rather than just in the sagittal plane. This would verify whether the positional sequence of joints and objects can be further refined and whether HMM training can be extended beyond the results obtained with bidimensional analysis. By doing so, HMMs can potentially support human-based diagnosis of motor patterns, with applications ranging from neural pathology disorders to the motor optimization of athlete performance.

## 5. Conclusions

The results show that the use of HMMs is useful for identifying changes in joint trajectory during the execution of the back squat exercise. Two-dimensional data of the trajectories, described by the joint range of motion, were sufficient for the HMMs to model movement and identify disturbances in the performed movements. These results recommend the application of HMMs to analyse the kinematic profile of the movement execution, allowing inferences about motor adjustments and disarrangements against a standardized reference.

The participation of human evaluators showed the consistency of the method with the professional interpretation of correct execution. Both the analysis of variance and the evaluators identified discrepancies in trajectories with increasing load. This finding shows that the natural variability of the exercise was not confused with altered movement patterns, but was interpreted as perceptible and as different motor adjustments from the reference. Not all identifications by the statistical model were perceived by human evaluators, but the HMMs were able to identify changes in the movement trajectory/pattern that were visually imperceptible to human observers.

Notably, this research presented intrasubject comparison results. Both the HMMs and the human evaluators used the subject themselves performing the exercise without load influence as a reference. Future work will provide a single model against which all volunteers will be compared.

## Figures and Tables

**Table 1:** Mean values of the maximum displacements obtained for each marker (barbell, hip, knee, and ankle) without load (0%) and with a load of 50% and 75% of body weight in each of the three executions for each participant.

	Load—0%				Load—50%				Load—75%			
Volunteer	Barbell	Hip	Knee	Ankle	Barbell	Hip	Knee	Ankle	Barbell	Hip	Knee	Ankle
01	56.0	26.5	8.1	0.5	59.6	32.8	8.8	0.3	56.0	31.2	7.8	0.4
02	62.5	37.5	8.2	0.2	56.0	35.3	7.4	0.2	55.6	33.9	6.9	0.2
03	65.3	40.3	11.5	0.6	65.6	41.7	11.5	0.7	62.3	38.0	10.5	0.6
04	63.6	25.6	8.1	1.3	66.9	31.0	9.4	0.9	63.2	32.5	9.8	1.0
05	63.0	28.2	7.4	0.6	67.1	35.2	8.8	0.5	69.1	37.2	9.7	0.5
06	54.1	25.5	8.0	0.3	58.0	31.9	9.2	0.2	58.5	31.5	9.5	0.2
07	79.3	39.5	10.8	0.2	75.5	34.2	9.5	0.3	65.8	26.0	7.3	0.2
08	59.0	31.5	8.0	0.3	63.5	36.2	9.5	0.4	64.6	37.8	9.8	0.6
09	72.7	38.6	11.0	0.4	75.7	44.4	12.0	0.4	79.3	46.7	13.6	0.6
10	46.7	29.9	7.2	0.5	49.0	30.9	8.7	0.4	50.0	31.8	8.1	0.5
Mean ± SD	62.2 ± 9.2	32.3 ± 6.1	8.8 ± 1.6	0.5 ± 0.3	63.7 ± 8.4	35.4 ± 4.5	9.5 ± 1.3	0.4 ± 0.2	62.4 ± 8.2	34.7 ± 5.6	9.3 ± 2.0	0.5 ± 0.2

SD: standard deviation. The values are measured in centimetres (cm).

**Table 2:** Minimum values for the number of states (N) needed in the HMMs to recognize the differences between the executions with different loads in cases of significant differences (p) per participant.

			Volunteers									
	Loads	p/n	01	02	03	04	05	06	07	08	09	10
Knee	0–50%	p	…	…	…	…	0.000	0.028	…	0.001	0.021	0.009
		n					30	40		20	30	20
	0–75%	p	0.901	0.007	0.035	0.023	0.000	0.013	0.002	0.000	0.000	…
		n		30	25	25	25	25	30	30	30	
Hip	0–50%	p	0.024	…	…	0.003	0.003	0.000	…	0.033	0.003	…
		n	30			20	40	45		35	30	
	0–75%	p	…	…	…	0.001	0.001	0.001	0.004	0.009	0.000	…
		n				20	35	45	20	35	35	

The three points (...) indicate that no significant difference was found by ANOVA.

## Data Availability

The data that support the findings of this study are available from the last author (dalton.pessoa-filho@unesp.br) upon reasonable request.
